# TPMS-based auxetic structure for high-performance airless tires with variable stiffness depending on deformation

**DOI:** 10.1038/s41598-024-62101-3

**Published:** 2024-05-19

**Authors:** Do-Yeon Kim, Hong-Seok Kim, Sarath Suresh Kamath, Xiangying Hou, Jae-Won Choi, Sang-Hu Park

**Affiliations:** 1 Graduate School of Mechanical Engineering, Pusan National University, Busan, 46241 Korea; 2 Department of Mechanical Engineering, The University of Akron, Akron, OH 44325 USA; 3 National Key Laboratory of Science and Technology On Helicopter Transmission, Nanjing University of Aeronautics, Nanjing, China; 4 School of Mechanical Engineering, Pusan National University, Busan, 46241 Korea

**Keywords:** Auxetic spoke structure, Triply periodic minimal surface (TPMS), Airless tire, Additive manufacturing, Copolymer rubber printing, A rotated primitive-type auxetic structure (RPAS), Engineering, Materials science

## Abstract

A novel auxetic structure applicable to airless tire spokes is designed based on the primitive-type triply periodic minimal surface (P-TPMS) to have higher stiffness through deformation under compressive force. For becoming higher stiffness by deformation, an unit cell of auxetic structure is proposed and its characteristics according to design parameters are studied. Based on the parametric study, a rotated primitive-type auxetic structure (RPAS) is designed, and the deformative behaviors of an airless tire with the RPAS spokes are compared with a generally used honeycomb spoke. Simulation and experiment results show that the designed RPAS tire exhibits more stable behavior through higher rigidity depending on the deformation state when compressed on flat ground and obstacles. This variable stiffness characteristic of RPAS tires can be advantageous for shock absorption and prevention of large local deformations. Also, the manufacturability of the designed auxetic structure is evaluated using real rubber-based additive manufacturing processes for practical application in the tire manufacturing industry.

## Introduction

Lattice structures are widely used for various structural design with advantage of their tunable geometries which allow engineers to realize high strength and lightweight mechanical parts^1,2^. And negative Poisson’s ratio (NPR) induces lattice structures to have counter-intuitive mechanical behavior of lateral contraction to a vertical load^3^, which imparts high strain energy absorption^4^, high damping performance^5^, and high resistance to indentation^6^, impact^7^, and fracture^8^. The lattice structure with NPR was named as auxetic structures by Evans^9^. Its unique mechanical characteristics are promising for higher protection and impact absorption of various devices. The application includes helmets^10^, automobile jounce bumper^11^, sensors^12^, and airless tires^13^.

The mechanical behavior of auxetic structures is highly dependent on their geometry. Various geometrically driven mechanisms inducing auxeticity have been classified typically into re-entrant, chiral, and rotating units^14,15^. Recently, triply periodic minimal surface (TPMS), which is precisely expressed by mathematical functions, has been considered as a promising way to generate the auxetic structures due to its excellent flexibility and controllability from the first proposed by Neovius^16^ and Schwarz^17^. However, TPMS is of relevance in natural things. It is observed as biological membranes^18^, equipotential surfaces in crystals^19^, and it has also been of interest in art, architecture, and others. Since TPMS structures have smooth surfaces and interconnections^20,21^, it allows to design a complicate three-dimensional (3D) shape without supports for high additive manufacturability^22^. Also, TPMS-based design with excellent mechanical functions in engineering applications has been reported such as a sandwich panel^23^, bone implant^24,25^, beam^26^, and thin-walled tube^27^. Some TPMS structures have a negative Poisson’s ratio. Soyarslan et al.^28^ investigated auxeticity of the four representative TPMS structures of primitive-, diamond-, gyroid- and I-WP-types. They found that only diamond-type TPMS structures with a specific range of volume fraction exhibit auxeticity. Yuan et al.^29^ reported that crossed layers of parallels (CLP)-type TPMS structures exhibited substantial auxetic behaviors with a Poisson’s ratio of − 1.14 at a compressive strain of 20%. However, most TPMS structures generally do not exhibit auxetic behaviors.

Numerous types of TPMS structures have the potential to possess auxeticity attributed to their tunable geometries. But, in order to obtain auxetic behaviors of desired TPMS structures, some modifications should be applied to them. The following studies modified primary TPMS structures to impart auxeticity, generating auxetic TPMS structures. Wang et al*.*^30^ modelled an auxetic tubular structure by slicing the cross-sections of a gyroid-type TPMS structure horizontally and then transforming the original orthogonal coordinates to cylindrical ones. Zheng et al.^31^ designed a buckling-induced auxetic structure by using a modified primitive-type TPMS (P-TPMS) equation with additional terms and investigated its varying stress–strain curves and Poisson’s ratios with respect to the relative density of the structures controlled by the level-set constant of the equation. Liu et al*.*^32^ designed a 3D auxetic structure by using a dual-period deformation function with Bezier fitting curves applied to a P-TPMS equation. The resultant structure consisting of rotated P-TPMS unit cells exhibited auxetic behaviors that could be controlled by adjusting different design parameters. The reported ideas to impart auxeticity for designing auxetic P-TPMS structures are to induce rotation of unit cells, causing alternating elliptical void patterns being closed under compression. Because P-TPMS structures have an array of circular voids, they have the potential to be transformed to rotation-induced auxetic structures. Furthermore, due to its simple and symmetrical geometry, design strategies for conventional lattice structures are available to impart auxeticity.

One of the important applications of auxetic structures is the spoke structures in airless tires. Spokes are usually designed by using lattice structures such as honeycomb^33^, TPMS^34^, re-entrant^35^, and anti-tetrachiral^13^. Spoke structures replace the role of air, support the weight of automobiles, and provide structural durability and stability in the mechanical behavior of airless tires. The previous work written by authors of this work^36^ demonstrated that the airless tire designed with a spoke structure using P-TPMS cells exhibited higher mechanical stability in terms of linear force–displacement behavior under vertical compression compared to conventional airless tires based on beam or honeycomb structures. However, a tire with a single stiffness value can struggle to satisfy the diverse performance requirements. To ensure that the tire possesses different stiffness under various conditions, thereby possessing suitable mechanical performance, adopting an auxetic structure that exhibits contraction behavior during compression should be advisable. While maintaining the basic framework of P-TPMS geometry, introducing auxeticity to the P-TPMS cells by rotating the unit cell as a simple design modification strategy could enable the design of airless tires with variable stiffness.

In this work, a novel airless tire design is introduced to have variable mechanical stiffness during compressive deformation by incorporating an auxetic structure composed of P-TPMS unit cells. The design process for generating the structures, namely a rotated primitive-based auxetic structure (RPAS), is proposed. The auxetic and mechanical behaviors of RPAS are evaluated through numerical simulation and experiments. The effect of rotation angle $\theta$, which is the main parameter inducing the auxetic behaviors, is discussed. Based on the results of the parameter study, an airless tire with RPAS (RPAS tire) is designed and fabricated. The mechanical performances of the RPAS tire are compared with the airless tire with a honeycomb structure (honeycomb tire) which is widely used to design a tire spoke. Finally, RPAS unit is fabricated by a customized direct-ink write (DIW) 3D printing system using a copolymer rubber-ink in order to evaluate the manufacturability of the RPAS tire.

## Methods

### Lattice design

**Figure 1:**
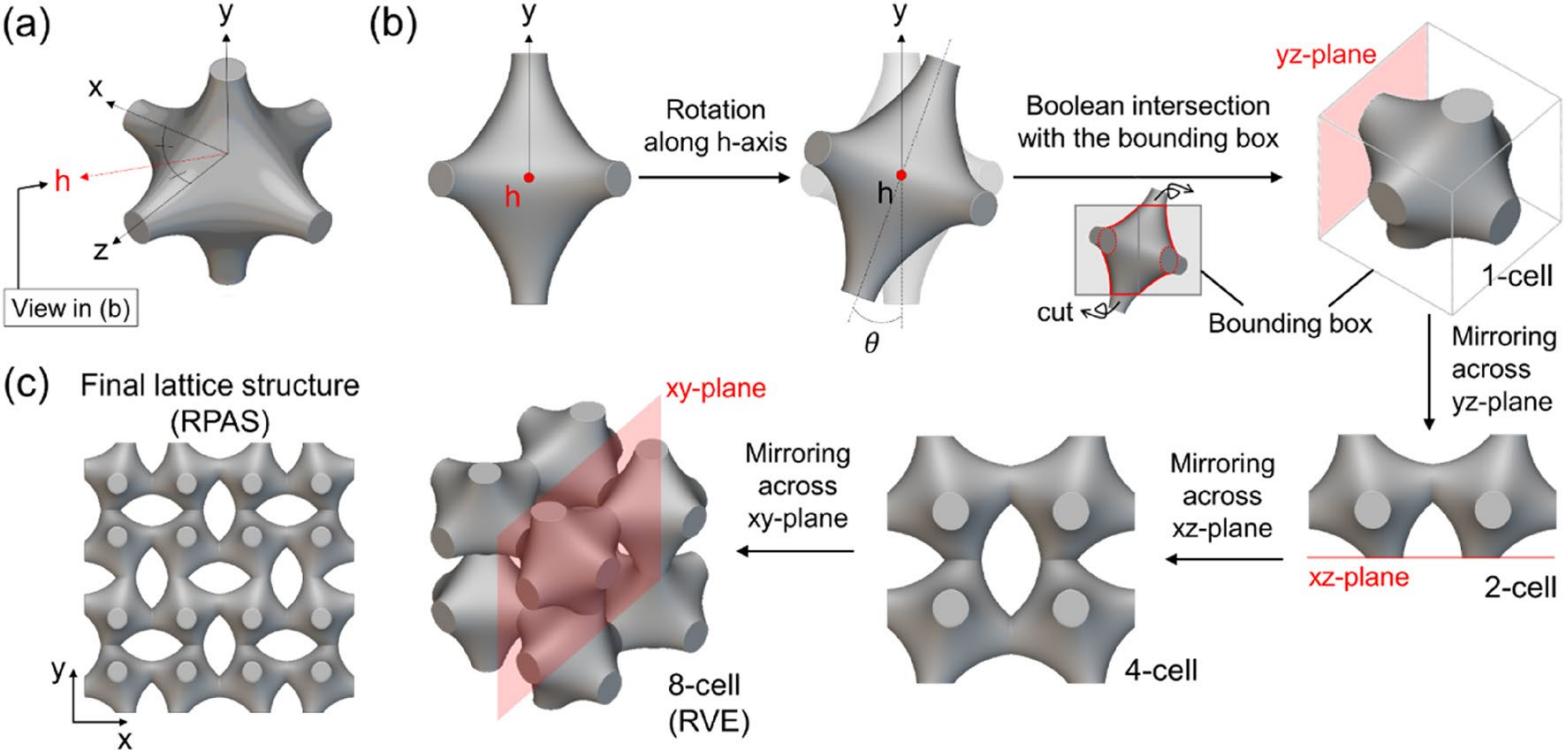
Design process of RPAS: (**a**) A Primitive TPMS unit cell generated by Eq. (1), (**b**) design of the representative volume element (RVE), and (**c**) a final lattice structure, rotated Primitive-based auxetic structure (RPAS) (software: nTopology, version 3.4, https://www.ntop.com).

The 3D auxetic structure using P-TPMS was generated on nTopology (Ver 3.4) referring to Eq. (1)^31^; P-TPMS (${\mathrm{\varnothing }}_{P}$) is defiened as,

1 $${\mathrm{\varnothing }}_{P}=(({\text{cos}}\left(2\pi x/L\right)+{\text{cos}}\left(2\pi y/L\right)+{\text{cos}}\left(2\pi z/L\right)-a({\text{cos}}\left(2\pi x/L\right){\text{cos}}\left(2\pi y/L\right)+{\text{cos}}\left(2\pi y/L\right){\text{cos}}\left(2\pi z/L\right)+{\text{cos}}\left(2\pi z/L\right){\text{cos}}\left(2\pi x/L\right))+c$$

where *L* indicates the unit cell length, and *c* is level-set constant which adjusts the volume fraction of the unit cell. The constant *a* controls how much the unit cell is deformed from the primary P-TPMS one, and *x*, *y*, and *z* is each coordinate value. The design process of RPAS proposed in this study is shown in Fig. 1a,b.

A P-TPMS unit cell with a unit length of 20 mm shown in Fig. 1a is rotated by $\theta$ along the *h*-axis, resulting oblique connections which would be bent when subjected to a compressive loading. Boolean intersection is then applied to the rotated unit cell with the bounding box of 14 $\times$ 14 $\times$ 14 mm^3^ in size as shown in Fig. 1b. The remaining volume (referred to 1-cell) after the intersection is a unit cell of RPAS. It is sequentially mirrored across the *yz*-, *xy*-, and *xz*-planes to generate a 2-cell, 4-cell, and 8-cell, respectively. The 8-cell structure is a representative volume element (RVE) of RPAS, and by repeating it in the three orthogonal directions, RPAS, which is a final lattice structure, is constructed as shown in Fig. 1c. The created RPASs are named according to $\theta$*.* For example, RPAS with $\theta$ = 15° is referred to as RPAS-15. Total five RPASs with $\theta$ = 0°, 5°, 10°, 15°, and 20° but identical *c*, *a*, and *L* (*c* = − 1.3, *a* = 0.4, *L* = 20 mm) are designed to investigate the effect of $\theta$.

### Numerical simulation

The deformation behaviors under compression of RPAS-0 to RPAS-20 were numerically investigated. The commercial software ANSYS (2020 R2) was used for the simulation with the implicit solver Static Structural module. The implicit solver requires less computation time than the explicit solver but has a limitation of self-penetration. In order to prevent self-penetration, the frictional contact conditions were defined on the contact faces of RPASs. An elastomer (named as Elastico supplied by Stratasys) was set as a constituent material of RPASs, and Yeoh 3rd hyperelastic model was used to fit the stress–strain curve of Elastico measured experimentally. The parameters of the model are shown in Table 1.

**Table 1:** Parameters of Yeoh 3rd order model for fitting the tensile stress–strain curve of Elastico.

Material constant (Pa)			Incompressibility parameter (Pa^−1^)		
${C}_{10}$	${C}_{20}$	${C}_{30}$	${D}_{1}$	${D}_{2}$	${D}_{3}$
76,363	295.96	29.067	0	0	0

**Figure 2:**
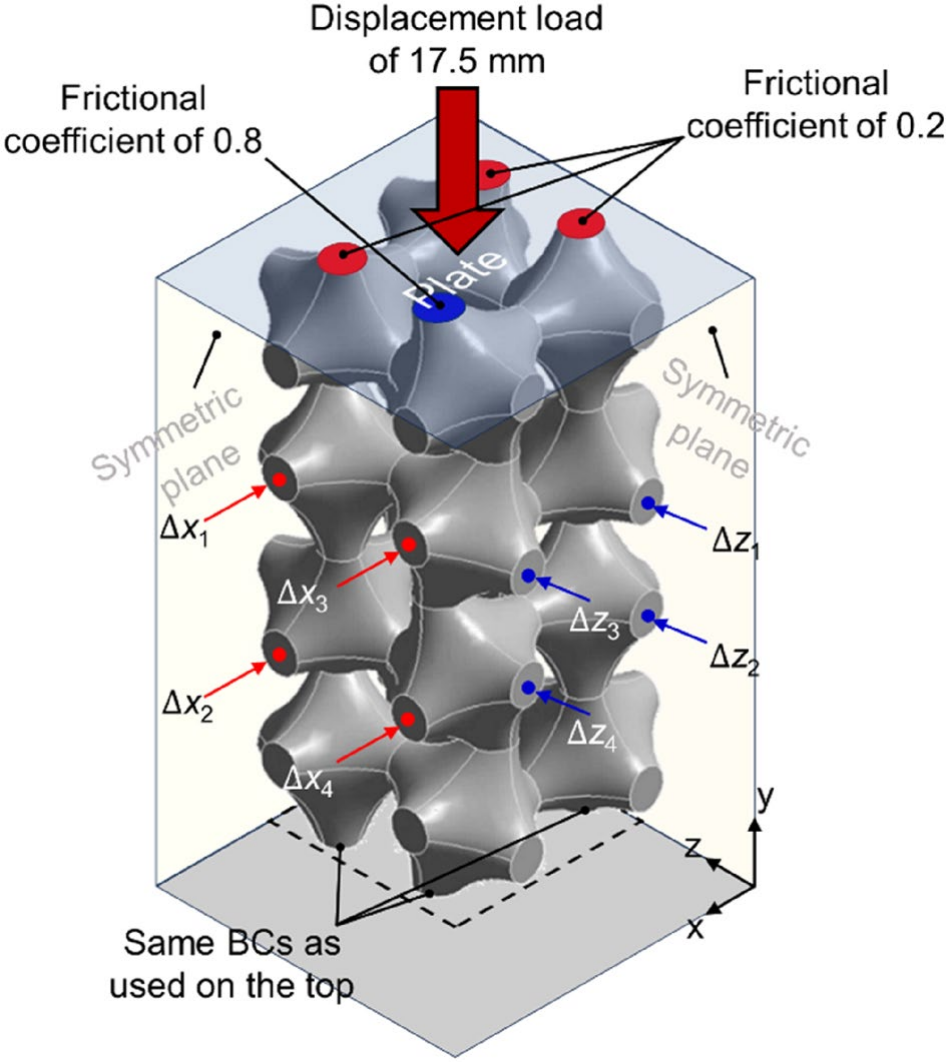
Boundary conditions for finite element analysis of a quarter RPAS model. The upper plate moves in negative *y-*direction while the lower plate is fixed. The frictional coefficients given on bottom and top are identical. *Δx* and *Δz* represent the contraction displacement of the nodes in *x*- and *z*-directions, respectively (software: ANSYS 2020 R2, https://www.ansys.com).

Compression simulations were conducted on one fourth models of RPASs with the boundary conditions explained in Fig. 2. The lower plate was fixed, and the upper plate was set to move by 17.5 mm in the negative *y* direction for the compression. Linear tetrahedral elements with a size of 0.8 mm were used to mesh RPASs after a convergence test. The displacements of the four nodes located on the centers of outermost faces of the two middle unit layers were recorded to calculate the Poisson’s ratio. Poisson’s ratio in *xy*-plane ${(\rho }_{yx})$ and *yz*-plane ${(\rho }_{yz})$ was calculated using the following equations:

2 $${\rho }_{yx}=-{\varepsilon }_{x}/{\varepsilon }_{y}=\Delta \overline{x }/\Delta y$$

3 $${\rho }_{yx}=-{\varepsilon }_{z}/{\varepsilon }_{y}=\Delta \overline{z }/\Delta y$$

where $\Delta \overline{x }=2\times \left(\sum_{i=1}^{4}{\Delta x}_{i}/4\right)$, $\Delta \overline{z }=2\times \left(\sum_{i=1}^{4}{\Delta z}_{i}/4\right)$ and $\Delta y$ is the vertical displacement.

An L9 orthogonal array was constructed as shown in Table 2. Each design parameter ($\theta$, *a*, and *L*) has three levels. Vertical stiffness, energy absorption, and Poisson’s ratio were selected as the factors of performance evaluation. The energy absorption was obtained by integrating the force–displacement curve up to densification onset displacement (${d}_{d}$). Similarly, the Poisson’s ratio was calculated to be averaged over the displacement range from zero to ${d}_{d}$. Compression simulation with the boundary conditions depicted in Fig. 2 were conducted to obtain the mechanical responses for the nine RPAS models generated by using the combinations of design parameters shown in the L9 array.

**Table 2:** L9 orthogonal array with three design parameters including rotation angle ${\varvec{\theta}}$, shape constant *a*, and length of Primitive-based unit cell *L*.

Design parameter			Response		
$\theta$(°)	*a*	*L* (mm)	Vertical stiffness (N/mm)	Energy absorption (mJ)	Poisson’s ratio
0	0.3	18	1.62	48.5	− 0.190
0	0.4	20	3.56	191.4	− 0.150
0	0.5	22	4.55	310.0	− 0.024
10	0.3	20	2.78	101.6	− 0.210
10	0.4	22	4.64	283.7	− 0.055
10	0.5	18	1.70	77.7	− 0.320
20	0.3	22	4.38	162.1	− 0.063
20	0.4	18	0.96	54.4	− 0.220
20	0.5	20	2.08	82.9	− 0.270

Each parameter has three levels. Responses include vertical stiffness, energy absorption, and Poisson ratio.

### Experiment on RPAS

To obtain the stress–strain curve of Elastico, tensile tests were conducted with reference to ASTM D412. Five dumbbell-shaped tensile specimens were fabricated by a PolyJet 3D printer (J55 Prime; Stratasys, USA) and underwent tensile tests by a universal test machine (RB 301 UNITECH-T; R&B, Korea). The results of tensile tests are represented in Fig. S1 in the supplementary material. RPAS-0, RPAS-10, and RPAS-20 were fabricated using Elastico by the same 3D printer, and compression tests were performed on them to validate the results of the simulations by the same universal test machine. They were compressed by the loading plate at displacement rate of 1 mm/min under room temperature, and their deformation shapes were captured by a digital camera to calculate the variations of the Poisson’s ratio (refer to Fig. S2 and Eqs. 1, 2 in the supplementary material for the method of calculating Poisson's ratio using experimental data).

## Results and discussion

### Mechanical behavior of RPAS

**Figure 3:**
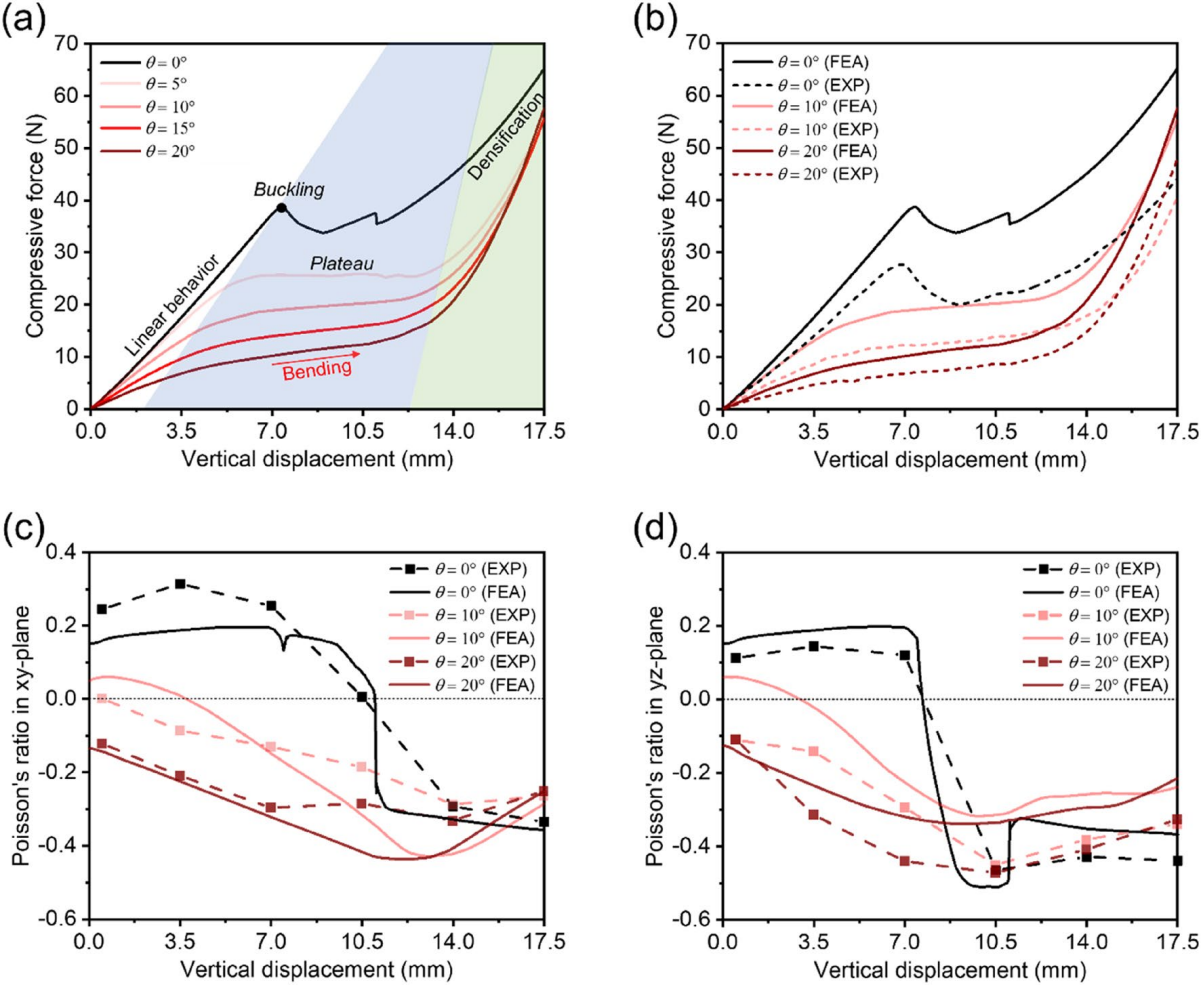
Variations of mechanical properties with respect to ${\varvec{\theta}}$: (**a**) Force–displacement curves of RPAS-0, -5, -10, -15, and -20. Comparison of (**b**) force–displacement, (**c**) Poisson’s ratios in *xy*-plane, and (**d**) Poisson’s ratios in *yz*-plane obtained from the numerical simulations and experiments.

In Fig. 3a, the force–displacement curves of RPAS-0 to RPAS-20 obtained through numerical simulations are depicted. The trend in the force–displacement curves varies depending on the deformation mechanism, whether it is stretching-dominant or bending-dominant. For the stretching-dominant structure, a post-yielding phenomenon occurs after the linear elastic region, which only RPAS-0 exhibited in this study. Buckling behavior causes negative stiffness with stress drop^37^, and multiple negative stiffness can be observed when a structure deforms gradually layer-by-layer^38^. Consequently, RPAS-0 showed the shortest plateau region. In contrast, RPAS-5 to RPAS-20 displayed no decrease in force, transitioning to a plateau region characterized by a nearly horizontal slope after the linear elastic region. As compression continues, RPASs enter the densification region where the force required for deformation rapidly increases, with RPAS-20 displaying the steepest slope compared to other RPAS at the same compressive displacement.

Figure 3b–d compare the results of numerical simulations with experimental results. While there is a discrepancy in force magnitude on the force–displacement curves, the overall trend remains consistent, indicating deformation behaviors were accurately predicted through numerical simulations. This disparity in force magnitude could be attributed to the moisture-sensitive characteristics of Elastico. It exhibits the property of becoming flexible when exposed to moisture for extended periods and becoming hard when dried. This resulted that the fabricated RPAS samples required less force to be compressed by the same displacement as the RPAS model in the simulation. The Poisson's ratio of RPAS is shown in Fig. 3c,d. In RPAS-0, similar to the post-yield behavior observed in the force–displacement curve, there is a region where Poisson's ratio rapidly decreases. For RPAS-10 and RPAS-20, Poisson's ratio gradually decreases and then increases after reaching a minimum value. Discrepancies between numerically obtained and experimentally measured Poisson's ratios are attributed to errors in measurement during video analysis.

**Figure 4:**
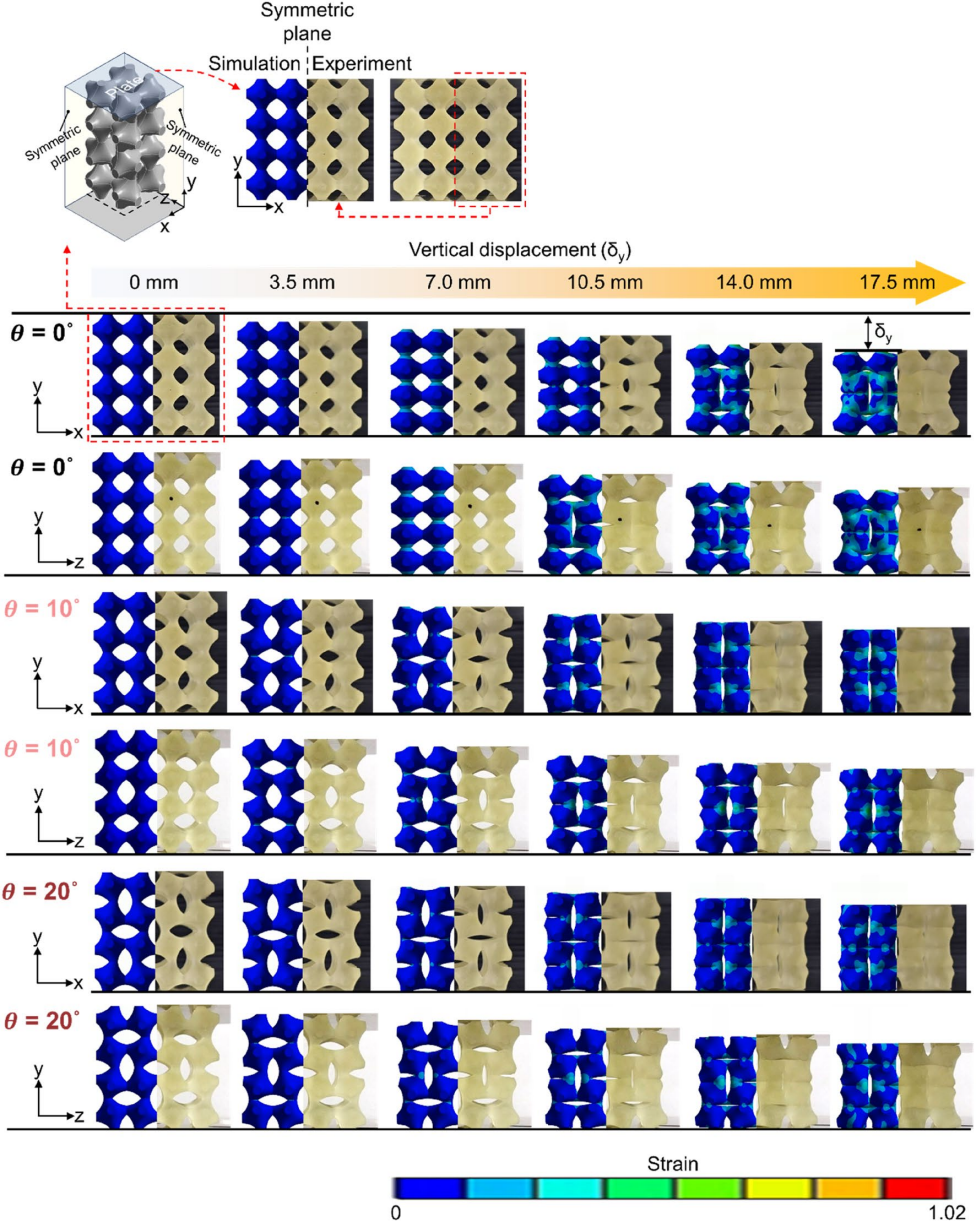
Deformation behaviors of RPAS-0, RPAS-10, and RPAS-20 under compressive loading force (software: ANSYS 2020 R2, https://www.ansys.com).

Figure 4 illustrates the compression deformation behaviors of RPASs in numerical simulations and experiments. The mechanical behaviors of RPAS according to compression displacement matches well. RPAS-0 shows no unit cell rotation even with a compression of 7 mm, unlike RPAS-10 and -20. As a result, as shown in Fig. 3c,d, RPAS-0 exhibits a positive Poisson's ratio without negative one until the displacement of about 7 mm. In contrast, RPAS-10 and RPAS-20 immediately undergo unit cell rotation after compression, exhibiting auxetic behavior. Differences were also observed in the symmetry of lateral contraction between RPASs with pre-rotated unit cells and those without. RPAS-0, showing auxetic behavior due to buckling, exhibited distinct lateral contraction only on the *yz*-plane at compression displacement of 10.5 mm. RPAS-10 and RPAS-20 did not show such asymmetrical contraction until reaching densification after compression. For RPAS with *θ* greater than 0, similar behavior was observed during compression, but there was a difference at the rate at which void volume between unit cells decreased. RPAS-20 with unit cells of higher pre-rotation value exhibited a smaller void volume than RPAS-10 at the same compression displacement, leading to an earlier emergence of densification as represented in the force–displacement curves in Fig. 3.

The distribution of strain exhibited variations depending on $\theta$. The strain distribution varied with $\theta$. As $\theta$ decreased, higher strain values were concentrated over a larger area because the deformation of the connections between unit cells became more severe at the same compression displacement. Specifically, at the compression displacement of 14 mm, RPAS-10 and -20 exhibited strain concentration only at the connections, while RPAS-0 showed strain concentration across the entire volume of the unit cells. This resulted in RPAS-0 exhibiting the highest reaction force at the same compression displacement.

**Figure 5:**
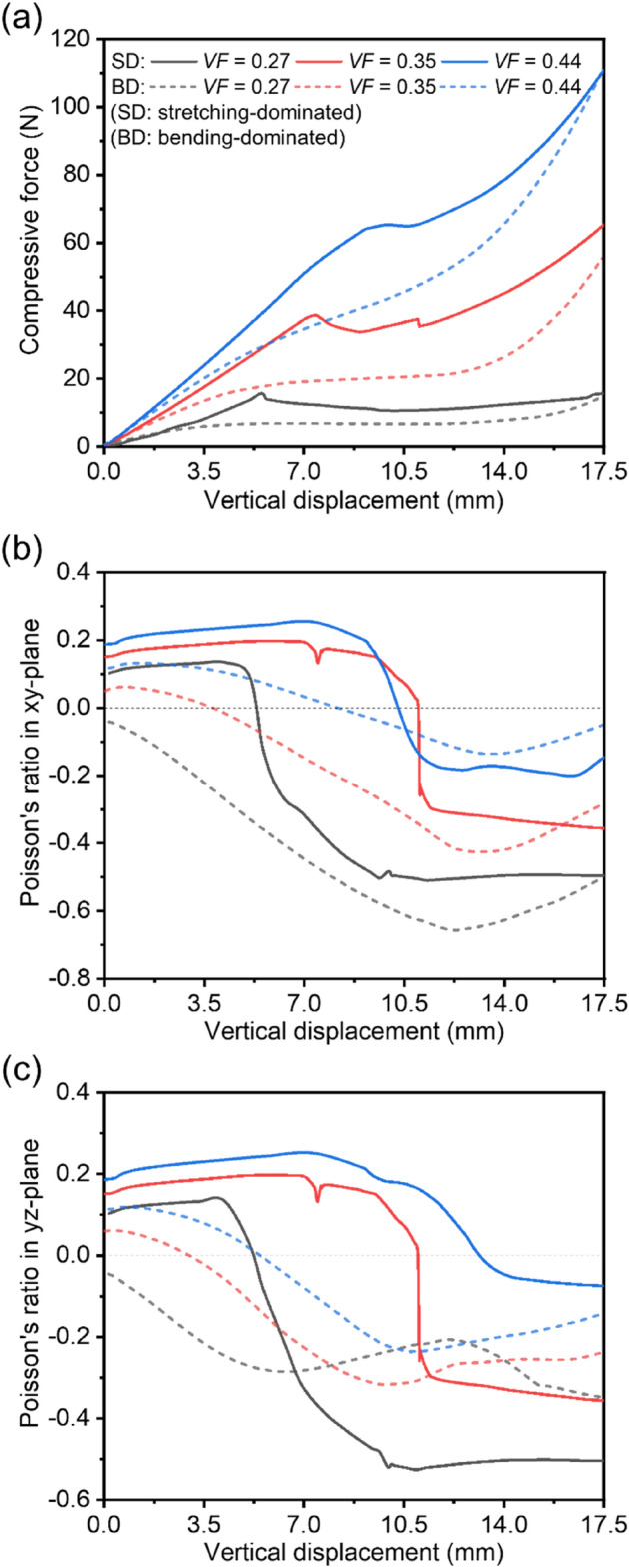
Mechanical behaviors of RPAS-0 s (stretching-dominant structure, SD) and RPAS-10 s (bending-dominant structure, BD) with three different volume fractions. (**a**) Force–displacement curves, (**b**) Poisson’s ratio in *xy*-plane, and (**c**) *yz*-plane.

As shown in Fig. 5, the mechanical responses of RPAS-0 and RPAS-10, each with three different volume fractions (*VF* = 0.27, 0.35, and 0.44 at *L* = 18 mm, *L* = 20 mm, and *L* = 22 mm, respectively) were investigated by simulation. The *VF*, which implies a volume fraction, has significant influence on the mechanical behavior of lattice structures, and the relationship can be described by a power-law function^39^. This equation not only predict the mechanical properties of the lattice structure, but also facilitates the identification of the deformation mechanism. The exponent of the power-law function approaches the value of 1 when the lattice structure is stretching-dominant, and approaches near 2 when its bending dominates. The stiffness of RPAS-0 (${K}_{R0}$) and RPAS-10 (${K}_{R10}$) in relation to each value of *VF* is represented by Eqs. (4) and (5), respectively.

4 $${K}_{R0}=29.0{(VF)}^{1.66}$$

5 $${K}_{R10}=34.2{(VF)}^{2.15}$$

This demonstrates that the stiffness of RPAS-10 is more sensitive to changes in the value of *VF* compared to that of RPAS-0, indicating that RPAS-10 is closer to a bending-dominant structure.

Based on Fig. 3 and Eqs. (4–5), RPAS-0 and RPAS-10 can be characterized as stretching-dominant and bending-dominant structure, respectively. As depicted in Fig. 5a, with decreasing the value of *VF*, RPAS-0 exhibits a lower stiffness, first peak force, and a shorter linear region due to earlier buckling, which are typical responses of stretching-dominant structure. Due to larger void spaces, densification occurs later for RPAS-0 with a lower *VF*, resulting in a longer plateau region and displaying a lower Poisson's ratio (see Fig. 5b,c). RPAS-10 also shows a lower stiffness, longer plateau region, and lower Poisson’s ratio when *VF* becomes lower.

### Effects of design parameters

**Figure 6:**
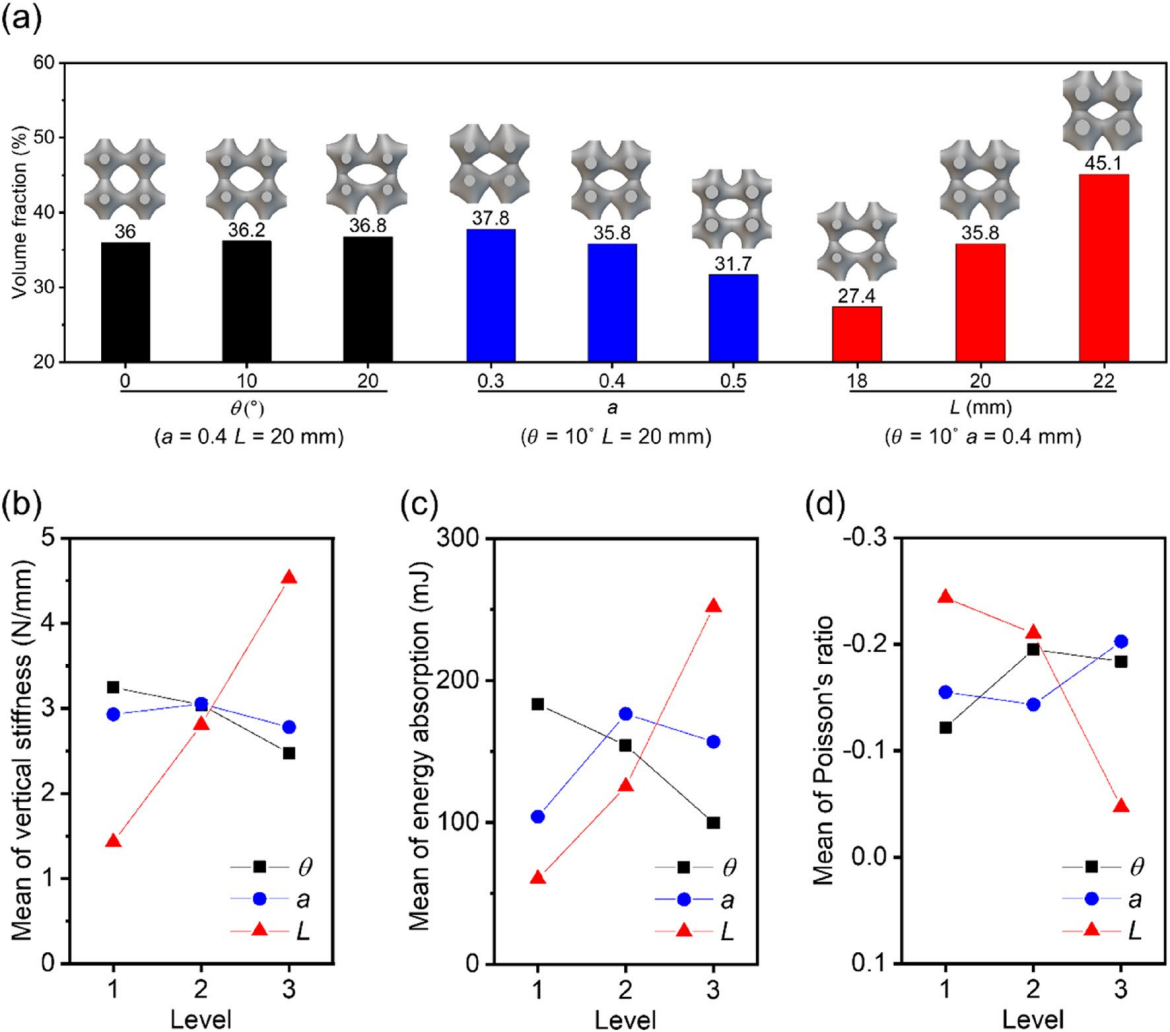
The *VF*s and mechanical properties of RPASs: (**a**) the variation of *VF* with respect to each design parameter. Effects of each design parameter on (**b**) vertical stiffness, (**c**) energy absorption, and (**d**) Poisson’s ratio (software: nTopology ver 3.4, https://www.ntop.com).

Figure 6a explores how the value of *VF* changes concerning different design parameters. The length of P-TPMS unit cell *L* significantly affects the *VF* because it determines the remaining volume in bounding box, while *θ* has minimal impact on the *VF* as the morphology of the unit cell remains almost same except its orientation. The *a* also influences the value of the *VF* because it changes the unit cell shape. In Fig. 6b–d, the main effect plot is shown depicting how design parameters affect the diverse characteristics such as vertical stiffness, energy absorption, and Poisson’s ratio. *L* emerges as the most influential factor on RPAS performance due to its significant impact on the amount of the material, showing trade-off between Poisson’s ratio and other properties.

The strengthened connections between unit cells and reduction in empty space hamper the rotation of unit cells, weakening the auxeticity of RPAS. *θ* notably influences the performances despite not altering the *VF*. *a* has a combined effect caused by both the volume fraction and shape change, so the mechanical properties do not change monotonically with respect to *a*. Based on these results, RPAS with *L* = 20 mm, *a* = 0.4, and *θ* = 10° was determined to be suitable for airless tire due to its negative Poisson’s ratio and moderate mechanical performances. The advantages derived from auxeticity can be utilized.

### Design and evaluation of airless tire

**Figure 7:**
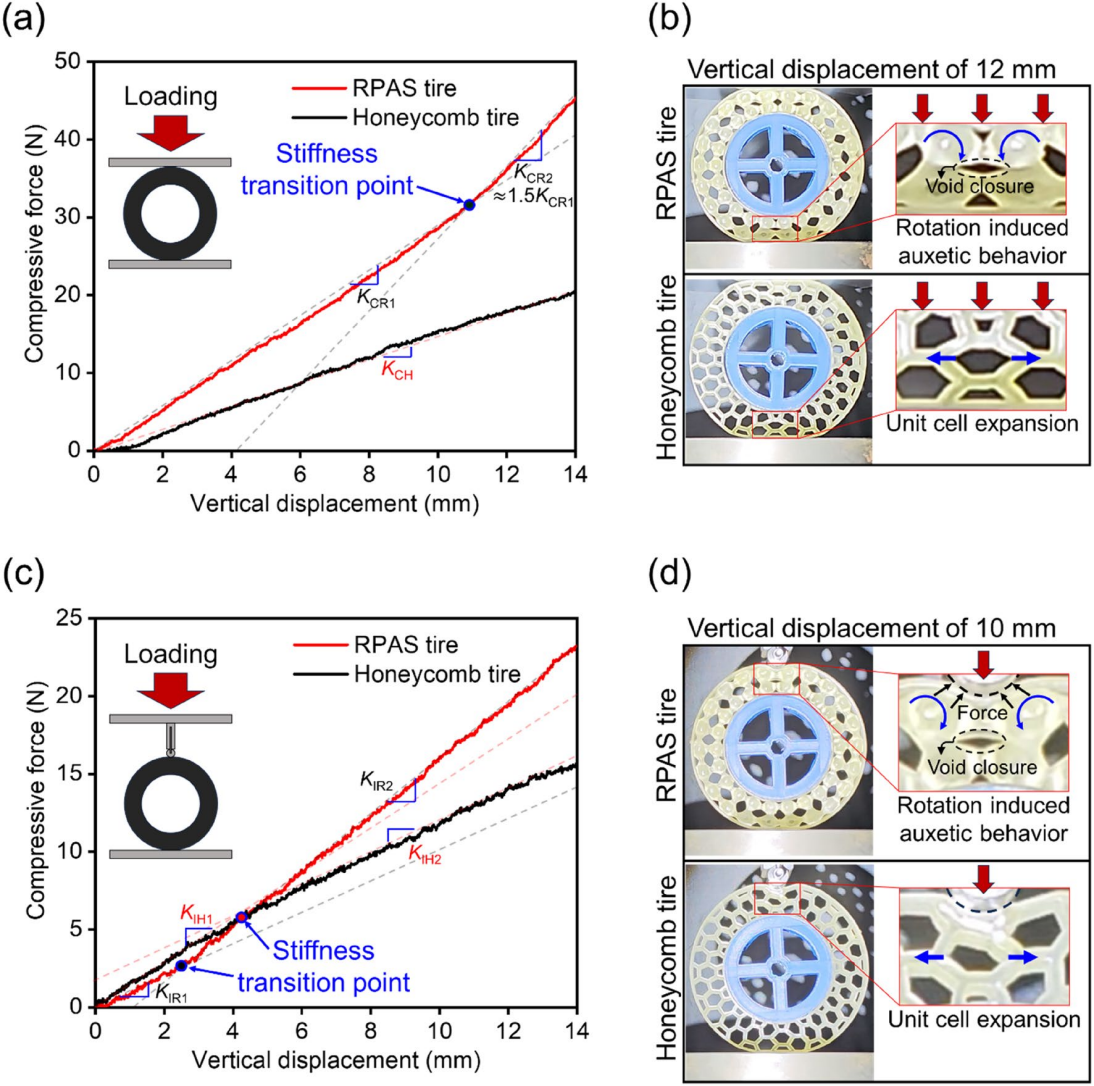
Results of compression tests: (**a**) force–deformation curves and (**b**) photographs of deformed tires under a flat compressive condition; (**c**,**d**) show those of test results under a small roller loading locally.

The modelling of the airless tire was conducted using nTopology. The RPAS (*θ* = 10, *a* = 0.4, *L* = 20 mm) is transformed into a spoke with 22 unit cells along the circumferential direction by converting the Cartesian coordinates to cylindrical ones. The honeycomb spoke is modeled using the method described in^36^, and its cell wall thickness and number of unit cells along the circumferential direction were set to 3.1 mm and 16. Each spoke was combined with the inner and outer rings whose diameters (thicknesses) are 108 mm (2 mm) and 162 mm (3 mm) respectively to create the airless tire, namely RPAS and honeycomb tires. Both tires had the same width of 28 mm and *VF* of 0.49. The airless tires were manufactured using the printer and material employed in creating the RPAS unit cell. The 3D printed RPAS and honeycomb tires had a nearly identical weights of 135 and 130 g, respectively, and were compressed at a displacement rate of 1 mm/min using the universal tensile machine. To investigate the deformation behavior of the tire when compressed against an obstacle, indentation experiments were also conducted. Instead of using an upper compression jig, a loading pin used in bending tests was employed to apply localized compression to the tire at a speed of 1 mm/min. Figure 7a–d depict the results of compression and indentation tests. As represented in Fig. 7a, it is observed that the stiffness of the RPAS tire is greater than that of a honeycomb tire of the same weight (*K*_CR1_ = 2.86 N/mm, *K*_CR2_ = 4.37 N/mm, *K*_CH_ = 1.46 N/mm).

As noted in authors’ previous study^36^, airless tires incorporating primitive structures exhibit high stiffness. Even when the unit cell is rotated to induce bending, the airless tire with the inherent high stiffness of the primitive structure achieves higher stiffness in its design compared to the stiffness of the widely used honeycomb structure. There is also a difference in the trend of the force–displacement curves. While the honeycomb tire shows nearly constant stiffness in force with compression displacement, the RPAS tire exhibits low stiffness (*K*_CR1_) until reaching the transition point, after which it has high stiffness (*K*_CR2_). This is attributed to the auxetic characteristics of RPAS. As shown in Fig. 7b, the auxetic behavior induced by rotation effectively reduces the void volume, thereby solidifying the structure.

Figure 7c depicts the force–displacement curves of the indentation tests. The trend of the force–displacement curves obtained from the indentation tests showed similarities with that obtained from the compression tests, but some differences were observed. At initial displacements of less than 4 mm, the resistance of the RPAS tire to local loads was lower than that of the honeycomb one. Additionally, stiffness transition points were observed in both tires. The stiffness of the RPAS tire was lower than that of the honeycomb tire up to a displacement of 2.6 mm (*K*_IR1_ = 1.08 N/mm, *K*_IH1_ = 1.38 N/mm), but beyond the transition point, the stiffness of the RPAS tire increased and surpassed the initial stiffness of honeycomb tires (*K*_IR2_ = 1.81 N/mm). The honeycomb tire exhibited a delayed stiffness transition compared to the RPAS tire, with a decrease in stiffness (*K*_IH2_ = 0.97 N/mm) observed after the transition point. These differences in stiffness variation are attributed to the auxetic properties of RPAS. Auxetic lattice structures exhibit high resistance to indentation as the material moves to where the load is applied^6^. In contrast, honeycomb structures with positive Poisson's ratio gradually weaken their resistance to indentation as the unit cells laterally expand with the progression of indentation. Figure 7d illustrates the difference in deformation behavior between the RPAS spoke and the honeycomb spoke under indentation loading.

**Figure 8:**
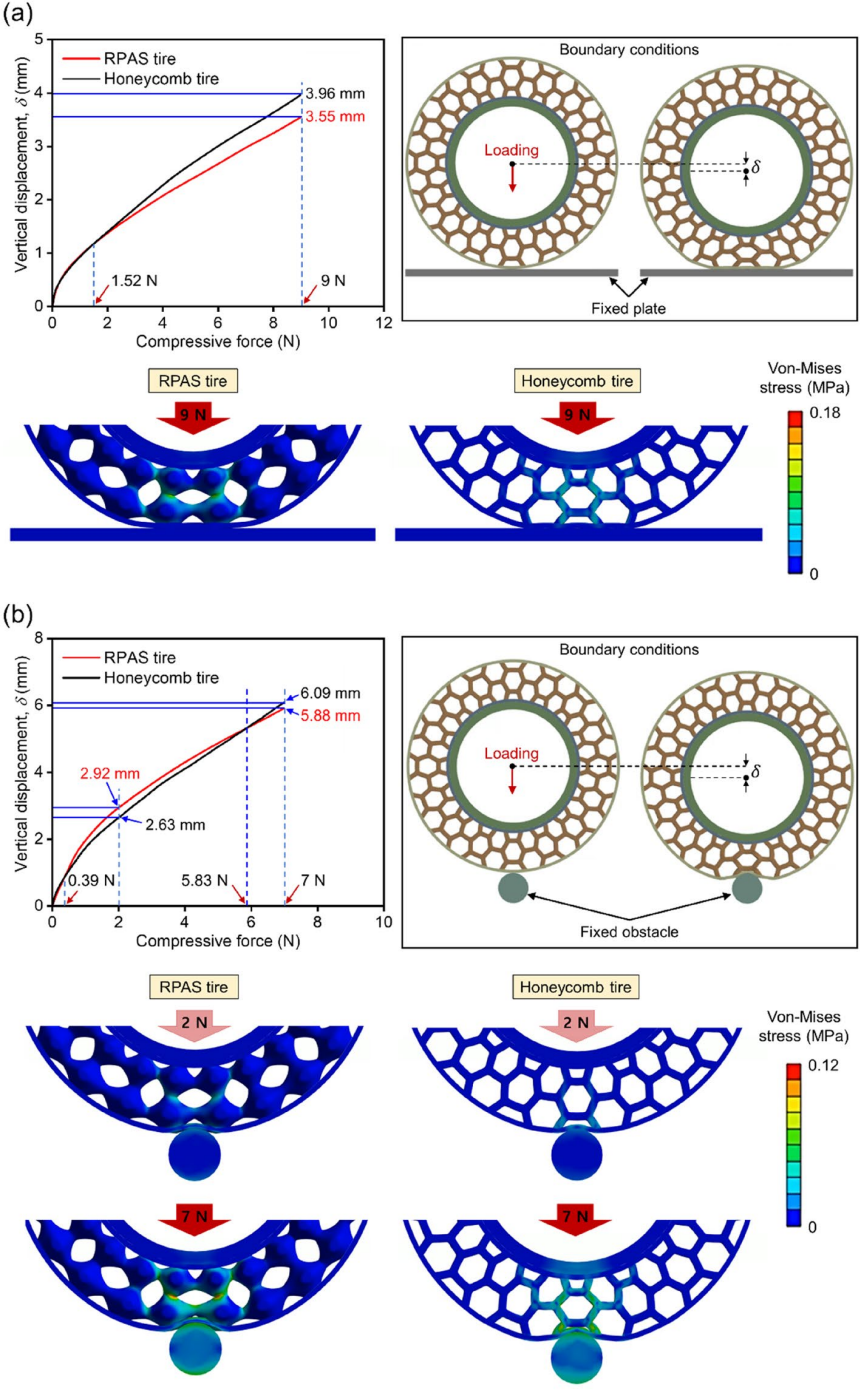
Comparison of simulation results of vertical deformation versus compressive force when tires passing over (**a**) flat ground and (**b**) obstacles.

The simulation results and analysis models are depicted in the inset of Fig. 8. It is shown in Fig. 8a that the deformation behavior of RPAS and honeycomb tires under compression load on flat ground. As shown in the displacement-force curves, except for the low-level load range below 1.52 N where primarily only the outer ring of both tires deformed, the RPAS tire exhibited similar vertical deformation comparing to that of the honeycomb tire under the low-level loads. However, the difference between two tires in a view of deformative behavior become larger with increasing load, reaching about 10% difference at 9 N. At this load, stress was concentrated on RPAS connections and inclined honeycomb cell walls due to their bending behavior, with stress propagating from unit cell connections to bulk in the case of the RPAS tire due to auxetic behavior, resulting progressive increase in resistance to deformation. Figure 8b compares the deformation behavior of compressed RPAS and honeycomb tires when passing over obstacles. Unlike the compression on flat ground, the RPAS tire deformed more than the honeycomb tire in the load ranges of 0.39 and 5.83 N. As shown in the graph at 5.83 N, the deformation amounts of both tires became equal, after which the deformation of the honeycomb tire increased rapidly further. In the case of RPAS, stress concentrated only on the connections at 2 N, but at 7 N, stress propagation to the bulk due to auxetic behavior significantly increased resistance to localized loads. In the case of honeycomb, stress propagated from inclined honeycomb cell walls near the outer ring to other horizontal and inclined cell walls. However, the variation of cross-sectional area was not as significant as in RPAS, resulting in less increase in resistance to deformation.

**Figure 9:**
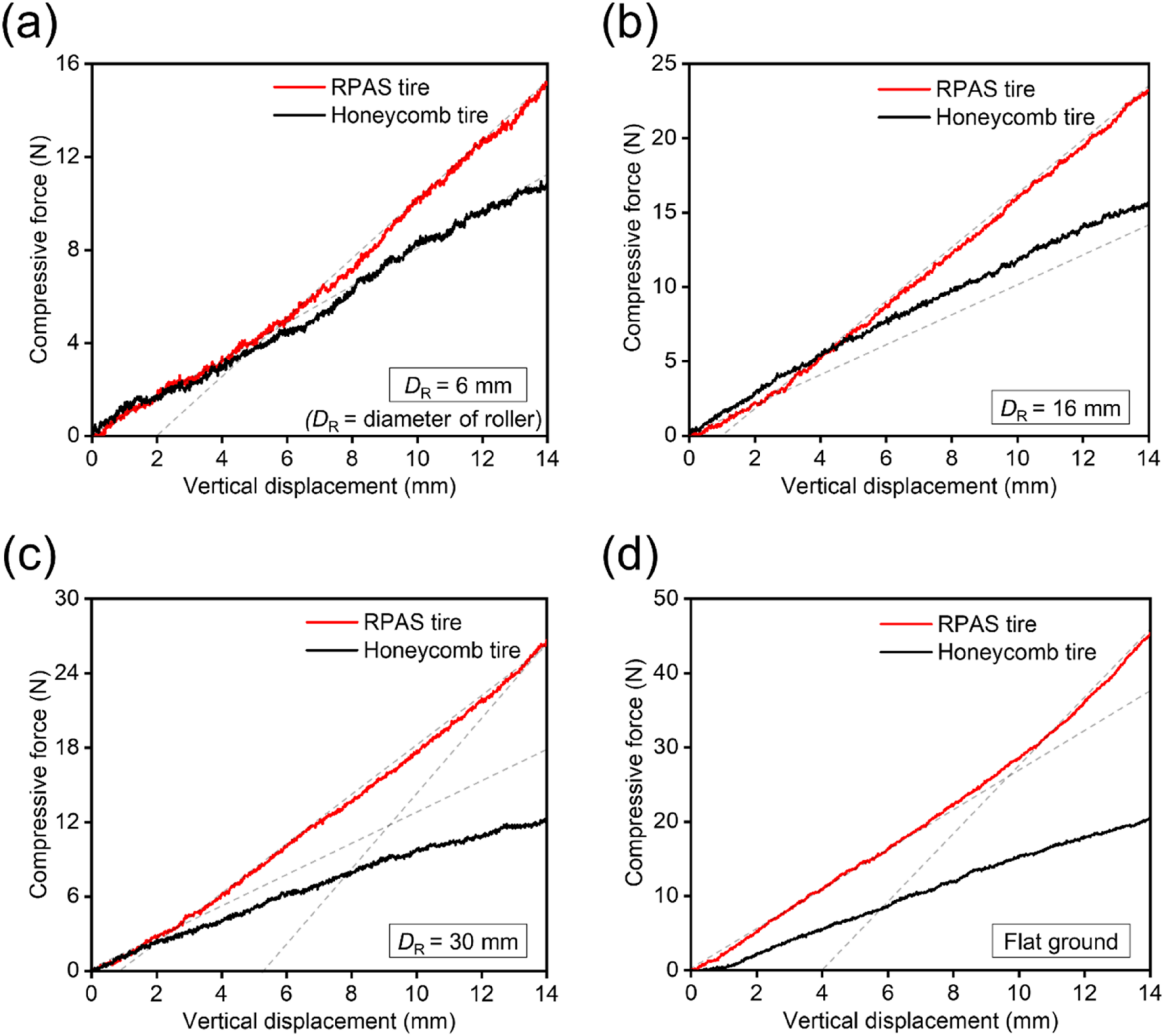
Compression test results: force–displacement curves of two tires locally deformed by rollers with different ${D}_{R}$: (**a**) ${D}_{R}$ = 6 mm, (**b**) 16 mm, and (**c**) 30 mm. (**d**) Force–displacement curves of two tires compressed on a flat ground.

Since the mechanical behavior of the airless tire is affected by the size of the obstacle, further indentation tests were conducted using two rollers of different diameters (6 and 30 mm). With a small ${D}_{R}$ (${D}_{R}$ = 6 mm), as represented in Fig. 9a, only the outer ring of the tire deforms significantly up to a compression displacement of about 4 mm, causing minor performance differences between the two tires compared to when ${D}_{R}$ = 16 mm (see Fig. 9b). As compression continues, the spokes begin to deform, leading to an increased gap between the two tire curves. For a large ${D}_{R}$ (${D}_{R}$ = 30 mm), deformation of the tire spokes starts immediately with the onset of compression, similar to the results of test on flat ground (see Fig. 9c,d). Therefore, the RPAS tire exhibits a higher resistance to deformation compared to the honeycomb tire from the beginning of compression. Additionally, at a large ${D}_{R}$, the spokes undergo more severe deformation at the same compression displacement compared to a small ${D}_{R}$, resulting in a larger gap between the two tire curves.

Most airless tires either exhibit a constant stiffness or experience a decrease in stiffness as the applied load increases. However, tires with a single stiffness value may pose disadvantages in terms of moderate shock absorption or load-carrying capacity (i.e., they can be too stiff or too flexible), making it difficult to achieve optimal ride comfort. Additionally, tires that experience a decrease in stiffness as the applied load increases may not be suitable for vehicles with large variations in load, such as trucks, as large deformation of the tire may occur due to significant loads. RPAS tires, leveraging auxetic properties, can offer solutions to these issues. They have low stiffness under small loads, allowing for easy deformation for shock absorption, while increasing stiffness under large loads to prevent excessive deformation of the tire. Moreover, based on the results of indentation tests and simulations, it can be expected that RPAS tires will exhibit appropriate mechanical performances depending on the size of obstacles encountered.

### Manufacturability test on RPAS

3D printable rubber inks were prepared based on the prior research^40,41^. Liquid styrene butadiene rubber (L-SBR) and UV curable isoprene (UC-102 M) employed in this study were sourced from Kuraray Company, Ltd. (Tokyo, Japan). 2-[[(butylamino)carbonyl]oxy]ethyl acrylate (BACA) as a monofunctional acrylate, trimethylolpropane triacrylate (TMPTA) as a trifunctional acrylate crosslinker, and stearic acid were obtained from Sigma-Aldrich (St. Louis, MO, USA). To enhance the rheological properties of the ink, fumed silica (Cab-OSil M-5) was acquired from Cabot Corporation (Boston, MA, USA), while Irgacure 819 as a photoinitiator was purchased from Ciba Inc., Switzerland. Vulcanizing agents and additives utilized in the study, including sulphur, zinc oxide (ZnO), tetramethylthiuram disulfide (TMTD), and N-cyclohexyl-2-benzothioazole sulfenamide (CBTS), were supplied by Akrochem Corporation (Akron, OH, USA). The copolymer rubber ink was prepared by first blending L-SBR and UC-102 M in the 1:1 ratio, in a high-speed mixer for 40 s at 2500 rpm. Followed by 12.5 phr BACA, 6.25 phr TMPTA with 4 wt% of a photoinitiator mixed with 10 phr of fumed silica for 1 min. To enable vulcanization 4 phr of ZnO, 1 phr of stearic acid, 4 phr of CBTS, 1 phr of TMTD and 2.5 phr sulphur were blended to homogeneity. The blended mixture's high viscosity makes it possible for the additives to diffuse evenly.

**Figure 10:**
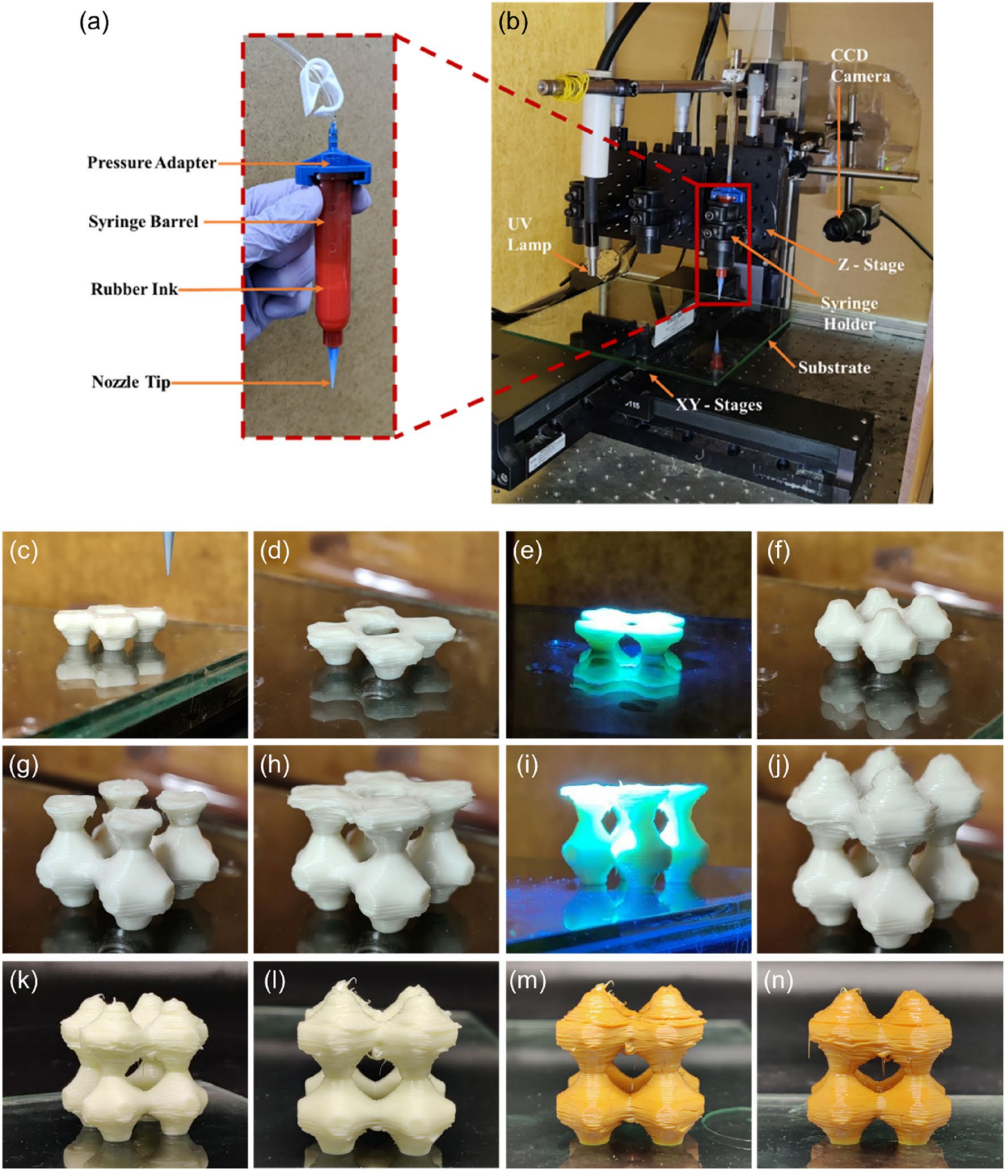
Direct-ink write (DIW) system setup: (**a**) close-up of the dispensing syringe; (**b**) customized DIW setup. Photographs of additive manufactured unit cells: (**c**–**n**) progressively printed results; (**e**,**i**) UV curing between layers; (**k**) and (**l**) green part; (**m**) and (**n**) part after vulcanization.

A customized DIW 3D printing system utilized in this study consists of several components, including a dispensing system comprising a pressure controller, syringes, and their holders, as well as a motorized *xyz*-stage as shown in Fig. 10a,b^42^. To ensure precise positioning control of the syringe tip, a PRO115 high-resolution XYZ linear stage (Aerotech, Inc.) was employed. Additionally, ThorLabs model XR35C/M manual translation stages were incorporated onto the Z-stage to calibrate the gap distance between the substrate and the dispensing tips. The extrusion process was facilitated by applying pressure through a Ultimus™ I pneumatic pump (Nordson EFD) connected to the syringe. For synchronization and control, G-code instructions were used to coordinate the movements of the XYZ linear stages and pressure controller, which were interfaced with LabVIEW software (National Instruments).

To prepare for 3D printing, a 3D model created using SolidWorks® CAD software was converted into an STL file format. Open-source software (Repetier-Host; Hot-World GmbH) was utilized to convert the STL file into layers and generate G-code instructions. These instructions contained the necessary toolpath and extrusion parameters for the 3D printing process. The layer thickness of 350 μm was adjusted based on a tip size. The print settings included a 100% infill density and a 45°/45° raster angle. To ensure the rubber ink remained in the optimal condition, each ink was promptly loaded into an Optimum® syringe barrel (Nordson EFD) after preparation, and remixed on the high-speed mixer for 30 s at 2500 rpm to eliminate any air bubbles in the material. Subsequently, the syringe was positioned on the XYZ stage and connected to the pneumatic pump in preparation for 3D printing. The ideal printing parameters were established by experimenting with various speed and pressure ranges.

In the present case, a traditional rubber vulcanization (e.g. used in the tire industry) is supplemented by a preliminary photocuring method that counts as an assisted dual cure system that happens during printing between layers. The process is represented in Fig. 10c–n. This technique helps hold the shape at narrow lattice sections, bridges, and overhangs. Additionally, traditional sulphur crosslinks were also introduced by utilizing additives including initiators, activators, crosslinking agents, and accelerants from the copolymer rubber ink. Vulcanization of the printed part was then achieved by thermal treatment of the sample (Fig. 10k, l) at 140–160 °C for 20 min. After thermal treatment, the sample was left to cool to room temperature. The vulcanized parts are shown in Fig. 10m,n. This feasibility study provides a compelling evidence on the realization of 3D-printed airless tires using rubber which is used in the current tire industry. 3D printing of full-scale airless tires is under investigation using modified rubber formulations which can satisfy various properties required for tires.

## Conclusions

Rotated primitive-based auxetic structure was newly designed for airless tire spokes with variable stiffness. The effect of design parameters (rotation angle $\theta$, shape constant *a*, and unit cell size *L*) on the mechanical properties of the RPAS were investigated by numerical and experimental methods. The optimal design parameters are obtained using the L9 orthogonal matrix, and the main findings are summarized as follows:

- Initial buckling-deformed spoke structure is converted to bending deformable 3D auxetic one by changing the rotation angle of the unit cells along a defined axis in the design process. The bending-induced auxetic structure shows more stable mechanical responses due to the gradual rotation of the unit cells.
- By adjusting $\theta$, the vertical stiffness, Poisson’s ratio, and energy absorption capacity of RPASs can be controlled. As $\theta$ increases, the stiffnesses decrease, and the auxetic property becomes more enhanced.
- RPAS and honeycomb tire were 3D printed, and their deformation behaviors under compressive loading were compared. The RPAS tire exhibited higher stiffness than the honeycomb one at the same weight and possessed variable stiffness with lower value at small displacement and higher value at large displacement. This can enhance ride comfort and improve the shock absorption and load-carrying capacity.
- The customized 3D printer with a DIW system was built, and the rubber-ink for 3D printing of RPAS was developed. The rubber RPAS was successfully printed without support structures for the manufacturing feasibility study.

### Supplementary Information

Supplementary Information.

## Data Availability

The datasets generated during and/or analysed during the current study are available from the corresponding author on reasonable request.
