# Supplementary material for: TPMS-based auxetic structure for high-performance airless tires with variable stiffness depending on deformation
Source: Sci Rep. 2024 May 19;14:11419. doi: 10.1038/s41598-024-62101-3 (PMC11102911; doi:10.1038/s41598-024-62101-3)
Supplement: Supplementary file 1 — Supplementary Information. [file 41598_2024_62101_MOESM1_ESM.docx]

# Tensile test
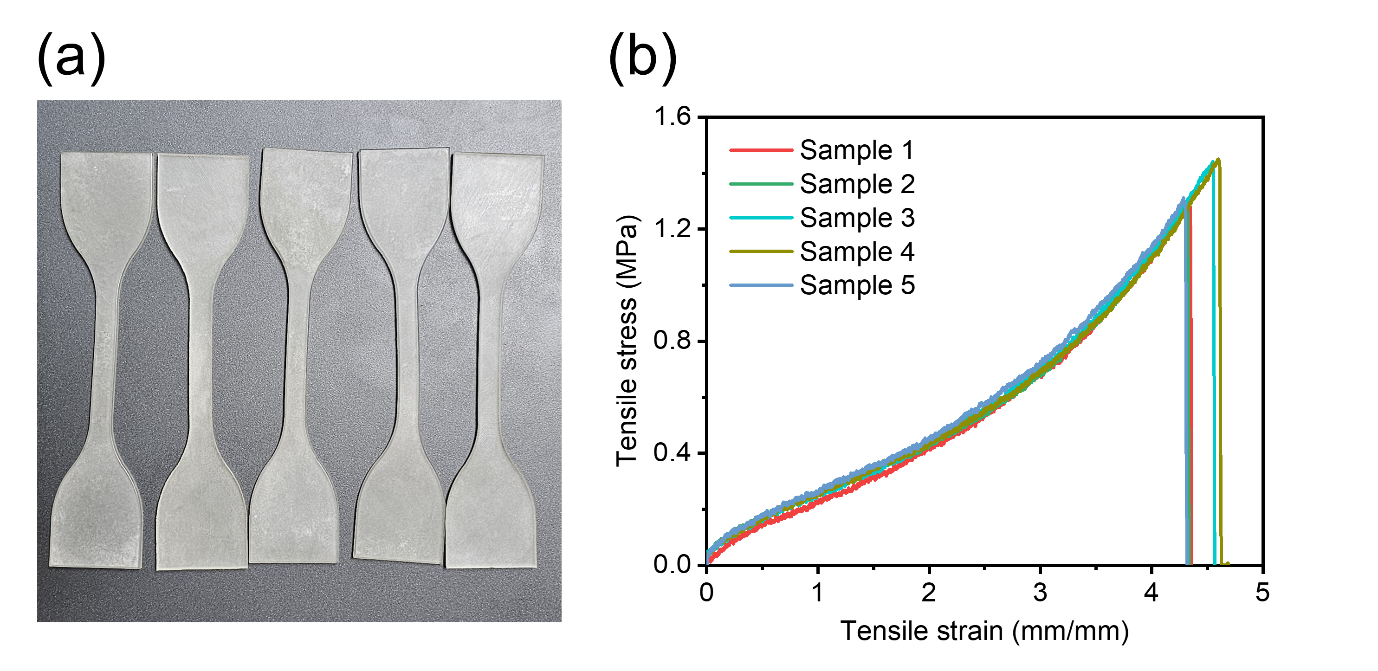


Fig. S.1. (a) 3D printed Elastico dumbbell-shaped specimens. (b) Tensile stress-strain curves of Elastico specimens.

# Poisson’s ratio measurement


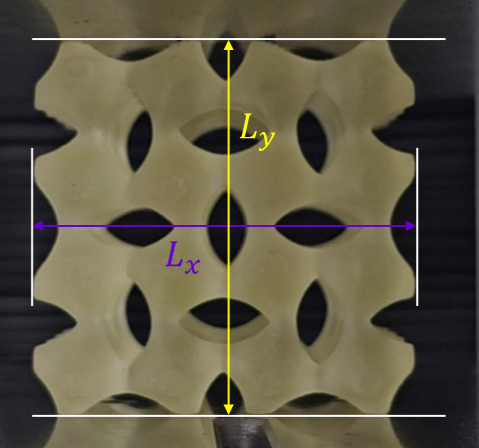


Fig. S.2. Definition of characteristic length for measurement of Poisson's ratio

The calculation of Poisson's ratio using experimental results is as follows:

$$-\frac{\frac{L_{x}-L_{0}}{L_{0}}}{\frac{L_{y}-L_{0}}{L_{0}}} (1)$$

$$-\frac{\frac{L_{z}-L_{0}}{L_{0}}}{\frac{L_{y}-L_{0}}{L_{0}}} (2)$$

# Effect of unit cell size to obstacle size ratio


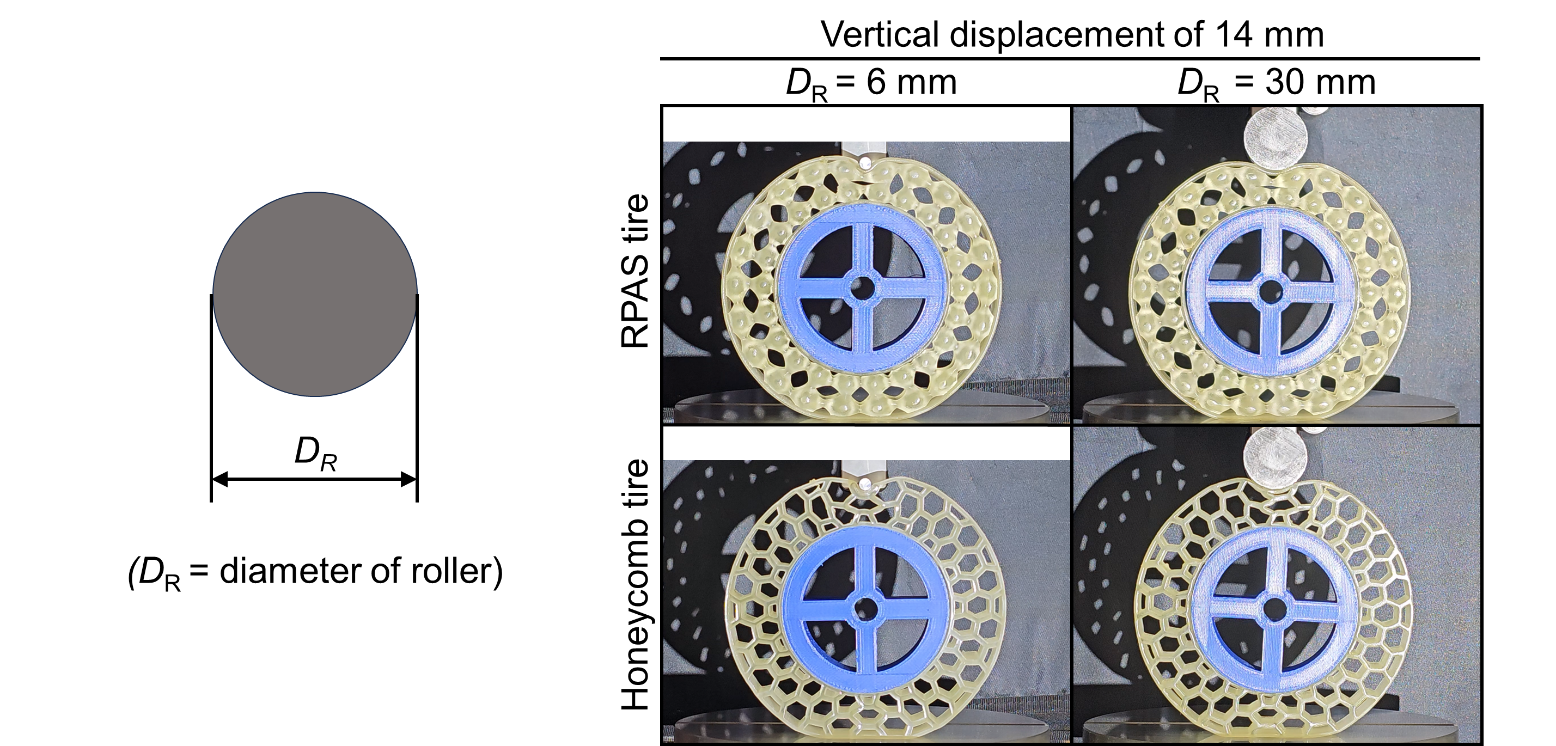


Fig. S.3. RPAS tire and honeycomb tire locally deformed by rollers with $D_{R}$ of 6 mm and 30 mm.

# Comparison of deformations between RPAS and honeycomb tires


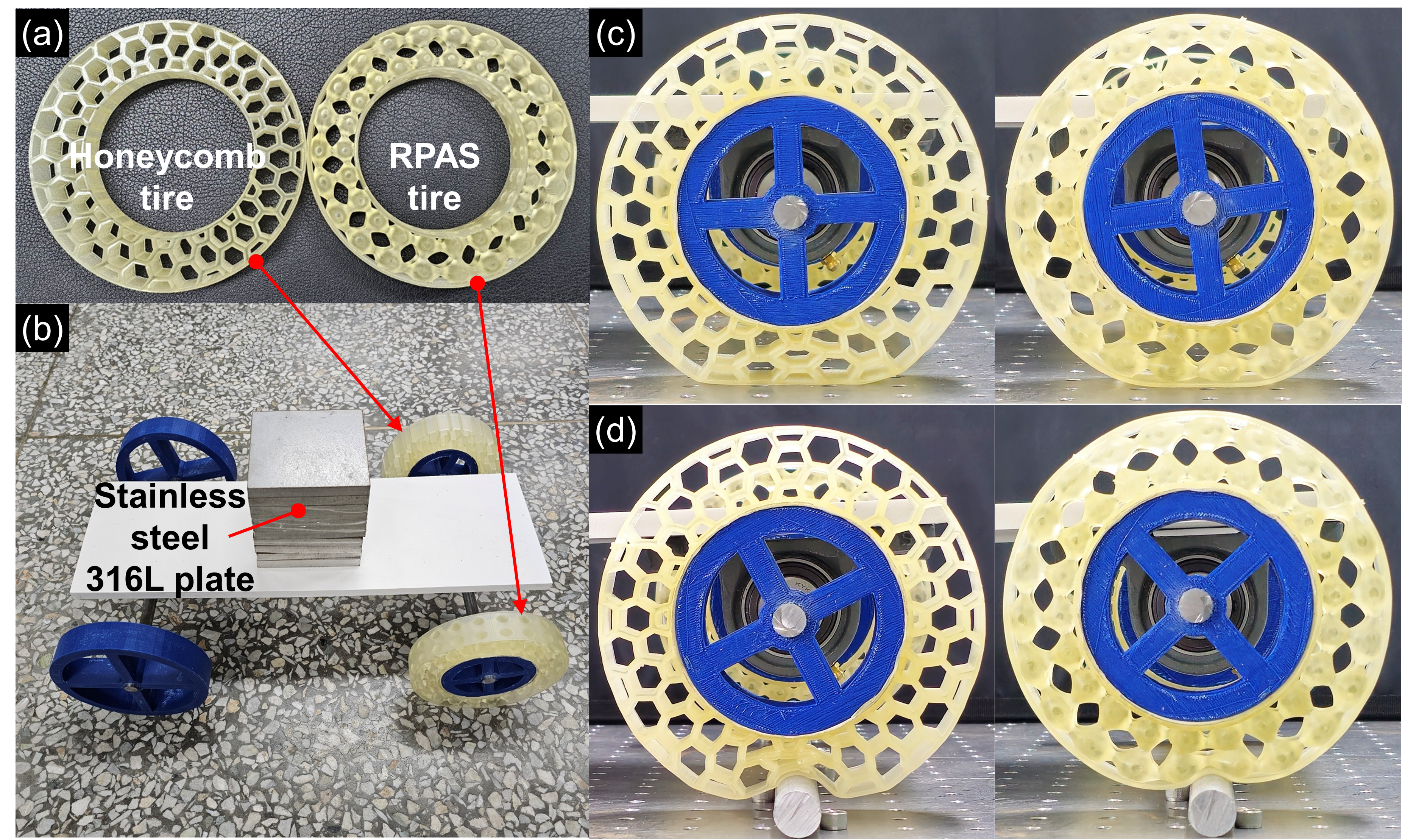


Fig. S.4. Comparison of deformation between RPAS tire and honeycomb tire. (a) 3D printed airless tires. (b) Customized cart equipped with the airless tires. To facilitate easier observation of tire deformation, a stack of ten stainless steel 316L plates was placed on the cart. Comparison of deformation between the honeycomb tire and RPAS tire: (c) deformed on flat ground and (d) deformed on obstacle.
